# A Biophysics of Epigenetic Rejuvenation

**DOI:** 10.3390/cells14161249

**Published:** 2025-08-13

**Authors:** Prim B. Singh

**Affiliations:** Department of Biomedical Sciences, School of Medicine, Nazarbayev University, 5/1 Kerei, Zhanibek Khandar Street, Astana 010000, Kazakhstan; prim.singh@nu.edu.kz

**Keywords:** epigenetic rejuvenation, age reprogramming, partial reprogramming, H3K9me3, H3K27me3, Flory–Huggins parameter, Hi-C, eAge, Shannon entropy

## Abstract

We present a synthesis based on epigenetics, machine learning and polymer physics from which emerges new relationships between the thermodynamic Flory–Huggins parameter (χ), epigenetic age (eAge) and Shannon entropy. Using a framework for the estimation of χ in the nuclear environment we show that $\chi \propto {eAge}^{-1}$ and $\chi \propto {Shannon\;Entropy}^{-1}$. As cells age, epigenetic drift results in “smoothing out” of the epigenetic landscape reducing the magnitude of χ. Epigenetic rejuvenation reverses epigenetic drift and restores χ to levels found in young cells with concomitant reduction in both eAge and Shannon entropy.

## 1. Introduction

### 1.1. Epigenetics: H3K9me3-Marked Heterochromatin-Like Domains/Complexes (HLD/Cs) and H3K27me3-Marked Polycomb-Group (PcG) Domains

**Figure 1:**
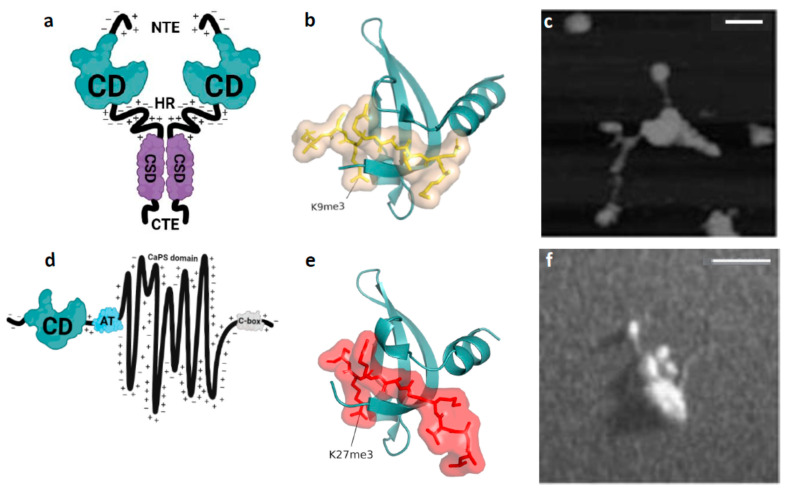
HP1β (aka CBX1, M31) and CBX2 (aka M33) proteins and their interaction with chromatin. (**a**) The functional domains of HP1β; all three mammalian HP1 isoforms (HP1α, β and γ) have similar functional domains [14]. HP1β contains two globular modules of around 30 Å in diameter, termed chromo-domain (CD) and chromo-shadow domain (CSD). The CD binds the H3K9me3 histone modification with an affinity in the μM range. The CSD mediates dimerization. The CD and CSD are connected by an intrinsically disordered hinge region (HR) and flanked by disordered N-terminal and C-terminal extensions (NTE and CTE, respectively). (**b**) The HP1βCD complexed with the H3K9me3 tail peptide (yellow stick). Binding of the H3K9me3 tail peptide causes the CD N-terminal region to draw upward and wrap around the peptide. (**c**) When HP1β is incubated with H3K9me3-marked oligo-nucleosome arrays a cluster or clutch of nucleosomes is observed. The image was visualized by scanning force microscope and taken from [11]. (**d**) Cartoon depicting the functional domains of CBX2. Towards the N-terminus of CBX2 is the CD that binds H3K27me3. There is a neighbouring AT-hook (AT) that can bind the minor groove of AT-rich DNA. The central portion of CBX2 is called the compaction and phase separation (CaPS) domain. It is an intrinsically disordered region with high net positive charge due to an abundance of lysine and arginine residues. CaPS domain gets its name from its ability to compact nucleosome arrays in vitro, to undergo liquid–liquid phase separation in vitro and to drive formation of phase separated puncta in nuclei [26]. The C- terminal “C-box” is required for incorporation of CBX2 into the PRC1 complex. (**e**) The CBX2 CD complexed with the H3K27me3 tail peptide (red stick). As with the binding of the HP1β CD to H3K9me3, binding of the H3K27me3 tail peptide causes the CBX2 CD N-terminal region to draw upward and wrap around the peptide. (**f**) When CBX2 is incubated with an oligo-nucleosome array assembled from HeLa histones a cluster or “clutch” of nucleosomes is clearly observed. Image was recorded using a transmission electron microscope and taken from [25].

Epigenetics is the study of heritable changes in gene expression that occur without altering the DNA sequence, including histone modifications, DNA methylation and non-coding RNAs [1]. Of the histone modifications, *tri*-methylated lysine 9 of histone H3 (H3K9me3) and *tri*-methylated lysine 27 of histone H3 (H3K27me3) are epigenetic [2]. H3K9me3- and H3K27me3-marked chromatin domains are macromolecular assemblies found in the genomes of most eukaryotes [3,4,5]. H3K9me3, along with the non-histone chromodomain (CD) protein heterochromatin protein 1 (HP1; Figure 1a), are hallmarks of constitutive heterochromatin in organisms as distantly related as fission yeast and humans [3,4]. HP1 “reads” the H3K9me3 modification through binding of the HP1 CD to H3K9me3 (Figure 1b; [6,7,8]). HP1 “readers” also dimerize via their chromo shadow domains (CSDs; Figure 1a) thereby conferring on HP1 dimers the ability to “bridge” H3K9me3-marked nucleosomes and form “clutches” of nucleosomes (Figure 1c; [9,10,11]).

Importantly, both H3K9me3 and HP1 are constituents of heterochromatin-*like* domains (HLDs) and smaller complexes (HLCs) that regulate chromatin template-dependent processes in the chromosome arms *outside* constitutive heterochromatin [12,13,14,15,16]. Hidden Markov model-defined H3K9me3 domains along the chromosome arms overlap with HP1-marked chromatin domains, there being ~20,000 to 30,000 HLD/Cs in the human genome, depending on cell type [17]. A genome-wide study that measured condensability at single-nucleosome resolution showed the strongest correlation is that of HP1-mediated condensability and H3K9me3-marked nucleosomes [18]. HLD/Cs contribute to the estimated 15.9% of the genome in human HCT116 cells that correspond to the heterochromatic (H3K9me3 and HP1α/β) chromatin state [19]. In the mouse, HP1β and H3K9me3 enrichments highly correlate across 42,890 H3K9me3-marked domains identified in embryonic stem (ES) cells [20]. HLD/Cs range in size from ~4Mb for HLDs to ~6kb for many of the HLCs [17]. Chromosomal regions assembled from H3K27me3-marked chromatin and Polycomb repressive complex 1 (PRC1)/Polycomb repressive complex 2 (PRC2) are termed Polycomb Group (PcG) domains [21]. The Chromobox 2 (CBX2) CD protein (Figure 1d) is a constituent of canonical PRC1 [5,22,23]. CBX2 “reads” H3K27me3 by binding of the CBX2 CD to H3K27me3 (Figure 1e; [24]). CBX2 does not “bridge” nucleosomes through homo-dimerization like HP1proteins. Instead, after CBX2 CD has bound H3K27me3, nucleosomes are “bridged” by the CBX2 compaction phase separation domain (CaPS domain; Figure 1d) that is able to compact a “clutch” of around four nucleosomes (Figure 1f; [25,26]). PRC2 contains the EZH2 histone methyltransferase that generates H3K27me3 [5,22,23].

**Figure 2:**
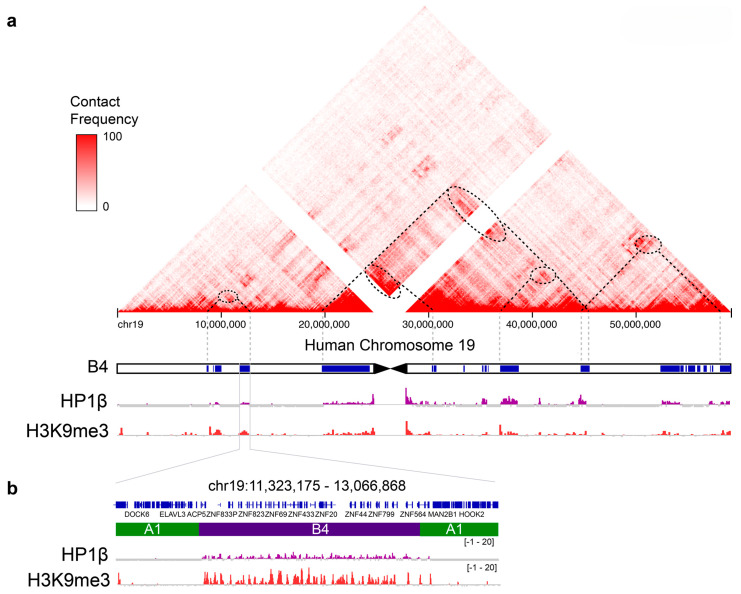
The heterochromatic B4 sub-compartment on human chromosome 19. (**a**) The triangle at the top shows the Hi-C map of chromosome 19. Contact frequency is given in red and greater intensity indicates greater contact frequency. Below the Hi-C map is the B4 sub-compartment given as blue “blocks”. The “blocks” overlap with H3K9me3-marked nucleosomes and HP1β enrichments that are given underneath the chromosomal map. The dotted lines start at “blocks” of the B4 sub compartment that interact and form contacts that emerge as enrichments in the Hi-C map (given as dotted ovals). (**b**) One of the “blocks” of the B4 sub-compartment is given at greater resolution, showing that HP1β and H3K9me3 enrichments overlap with the KRAB-ZNF repeats within the “block”. Modified from [15].

The above observations indicate that individual chromosomes have a “blocky” structure; chromatin along the chromosome arms in the interphase nucleus can be considered as a set of interspersed heterochromatic “blocks” and euchromatic “blocks” that are contiguous along linear chromosomes. HLD/Cs alternate with euchromatic “blocks” with some of the largest HLDs found at the KRAB-zinc finger (KRAB-ZNF) gene clusters on human chromosome 19, which are up to 4Mb in size [27,28]. Notably, Hi-C studies have identified a manually annotated heterochromatic B4 sub-compartment that emerges from contacts between the large KRAB-ZNF HLDs (Figure 2; [29]). Compared to HLDs, the vast majority of H3K27me3-marked PcG domains are small, in the region 4–5 kb, with less than 5% being greater than 50kb in size [30]. Like H3K9me3-marked HLD/Cs, H3K27me3-marked PcG domains contribute to the “blocky” nature of linear chromosomes. Homotypic interactions between H3K27me3-marked PcG domains contribute to contact enrichments that emerge as the B1 sub-compartment in Hi-C maps [29,31].

### 1.2. Machine Learning: Linear vs. Non-Linear Epigenetic “Clocks”

H3K9me3-marked HLD/Cs and H3K27me3-marked PcG domains are conserved chromatin-template dependent mechanisms that regulate the cell-to-cell (epigenetic) inheritance of gene activity states. Another essential epigenetic pathway involves the CpG (de)methylation machinery [32]. There is functional cross-talk between chromatin-template dependent and DNA (de)methylation pathways [33,34,35]. H3K9me3 “readers” act as adapters for the recruitment of heterochromatin-associated proteins [36,37] including DNA methyl transferases [38,39,40,41]. Most of the thousands of H3K27me3-marked PcG domains found in the genome overlap with CpG islands that are generally hypomethylated [42,43,44]. Hypomethylation is mediated by a functional interaction of PRC2 with Tet dioxygenases that demethylate methylated CpGs (meCpGs) [35,45,46]. Recent studies, to be described, have shown that as cells age chronologically, cross-talk between the two epigenetic pathways impacts age-dependent changes in CpG methylation at specific meCpGs. When these changes in meCpGs are inputted into mathematically derived age estimators, “epigenetic clocks”, the output accurately predicts epigenetic age (eAge) [47]. eAge is thought to be a measure of biological age [48].

**Figure 3:**
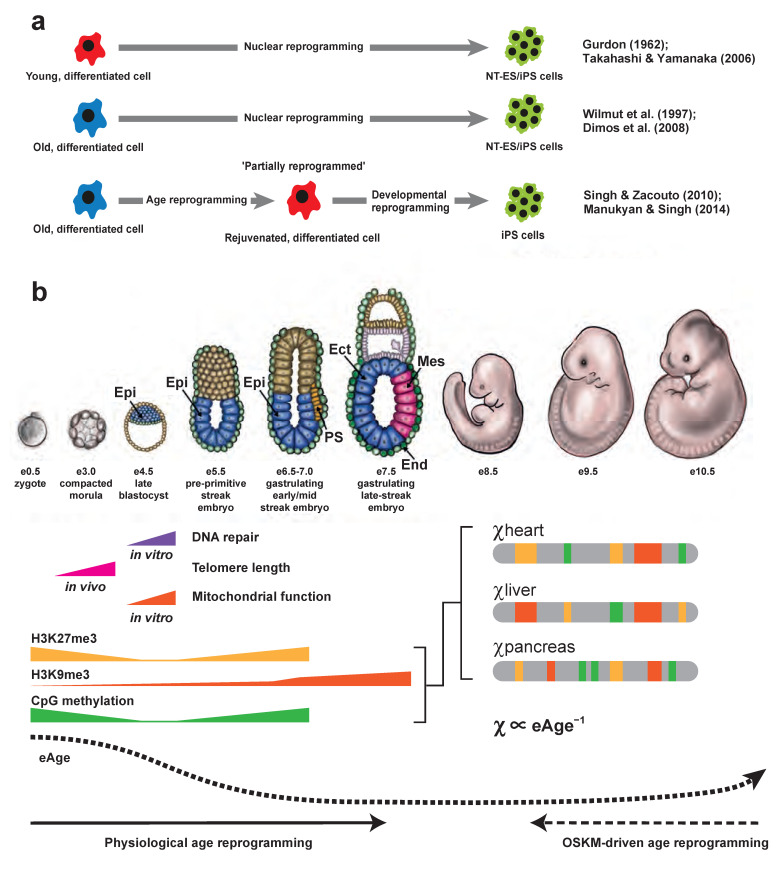
Age reprogramming, χ, eAge and the phylotypic progression. (**a**) Age reprogramming as a separable element of nuclear reprogramming. Top row, shows work of Gurdon (1962; [91]) and Takahashi and Yamanaka (2006; [92]). Using somatic cell nuclear transfer (SCNT) in *Xenopus laevis* it was shown that fully differentiated intestinal cells from feeding larvae could support the development of normal muscle and nerve cells after transplantation to enucleated eggs [91]. The differentiated cells underwent nuclear reprogramming through an embryonic (nuclear transfer embryonic stem cells; NT-ES cells) stage after SCNT. Work in mice showed that after introduction of *Oct4*, *Sox2*, *Klf4* and *c-Myc* “reprogramming factors” (OSKM) into embryonic fibroblasts or tail-tip fibroblasts from young male (7-week-old) and female (12-week-old) mice, the cells underwent nuclear reprogramming resulting in induced pluripotent stem (iPS) cells [92]. Middle row, shows the work on “Dolly-the-sheep” by Wilmut et al., (1997; [93]) and that on human iPS cells by Dimos et al., (2008; [94]). Dolly was the first mammal cloned from a cell from an adult animal. She was derived from a 6-year-old mammary gland epithelial cell by SCNT. A total of 29 of 277 reconstructed embryos that developed to the blastocyst stage (that contain NT-ES cells) were implanted into surrogate Scottish Blackface ewes [93]. One gave rise to a live lamb, Dolly. iPS cells were generated after introduction of OSKM reprogramming factors into skin fibroblasts collected from an 82-year-old patient [94]. Bottom row, shows the work of Singh and Zacouto (2010; [95]) and Manukyan and Singh (2014; [52]). It was hypothesized [95] that nuclear reprogramming could be separated into age reprogramming and developmental reprogramming. The test was to introduce OSKM “reprogramming factors” factors into an old cell (in blue) characterized for age-related markers and during the trajectory from old cell to iPS cell (in green), a search would be made for a stage where the marker(s) were reduced or lost, indicating rejuvenation (in red). Such “*partially reprogrammed*” cells, where OSKM expression is interrupted, would retain their specialized characteristics, i.e., not exhibit characteristics of embryonic cells. Using this experimental approach it was shown that age and developmental reprogramming are separable [52]. (**b**) Physiological age reprogramming: χ, eAge and the phylotypic progression. Physiological age reprogramming takes place during pre-implantation/early post-implantation development (dark arrow at bottom). During this period the hallmarks of ageing, telomere attrition, mitochondrial dysfunction, DNA repair and epigenetic drift are rejuvenated. Epigenetic rejuvenation of histone and DNA modifications during pre-/early-post implantation development results in a global repressive epigenetic landscape in or around gastrulation. Specifically, H3K9me3 (in orange) increases and becomes enriched over promoters, gene bodies and termination sites in mesodermal and endodermal cells on day E8.25 [96,97]. Global levels of H3K27me3 (in yellow) histone modification and CpG methylation (in green) reach maximal levels around day E6.5 [98,99]. Towards the end of gastrulation, as cells enter the phylotypic progression, the repressive epigenetic landscape undergoes a profound reorganization. There is loss of repressive modifications at lineage-specific genes and packaging of lineage-inappropriate genes into repressive H3K9me3-marked HLD/Cs and H3K27me3-marked PcG domains. As cells become committed to specific lineages, lineage-specific distributions of H3K9me3-marked HLD/Cs, H3K27me3-Marked PcG domains and DNA methylation are established, depicted along idealised chromosomes (in grey) from heart, liver and pancreas. H3K9me3-marked HLD/Cs and H3K27me3-marked PcG domains are newly assembled—not, as yet, subject to epigenetic drift—and the magnitude of χ for HLD/Cs and PcG domains in different lineages is at a maximum (χheart, χliver and χpancreas are shown). The establishment of “pristine” lineage specific epigenotypes having maximal χ values coincides with the eAge minimum [90,100], giving the relationship $\chi \propto {eAge}^{-1}$. Maximal levels of χ also coincide with the described epigenomic ground-state pattern established in the embryo [62]. OSKM-driven age reprogramming (dotted line at bottom) takes advantage of the natural process of physiological age reprogramming enabling experimental rejuvenation of hallmarks of aging in old differentiated cells. Epi, Epiblast; Ect, Ectoderm; Mes, Mesoderm; End, Endoderm; PS, primitive streak. Modified from [49].

Estimation of biological age is important because it enables the identification of genetic and environmental factors that impact the ageing process, as well measuring the efficacy of interventions that reverse ageing such as Oct4/Sox2/Klf4/c-Myc (OSKM)-driven age reprogramming, which rejuvenates hallmarks of ageing (Figure 3a; [49,50]). Age-dependent changes in the epigenome are termed epigenetic drift, which is a primary hallmark of aging [51]. Epigenetic rejuvenation can reverse epigenetic drift [52,53,54,55,56,57,58] and is a key driver of OSKM-driven age reprogramming. If epigenetic rejuvenation is stopped, rejuvenation of other hallmarks fails [55,59].

A widely used pan-tissue “epigenetic clock” is the Horvath clock [60] that is trained on chronological age using a penalized linear regression-based framework where a line of best fit is calculated through the meCpG levels at specific sites and chronological age. The learned relationship is used to predict eAge that provides a surrogate marker for biological age [48]. The “ticking” of epigenetic clocks is thought to be initially driven by age-dependent changes in CpG methylation in tissue stem cells—these changes being compounded by both gradual loss of tissue stem cells and amplification during differentiation of progeny, whose descendants go on to populate tissues possessing more advanced eAges [61,62]. Each tissue has its own rate of “ticking”, giving rise to tissues having different eAges [61]. A clue to the molecular basis of what drives the age-dependent changes in CpG methylation used by linear models to predict eAge comes from a survey of 59 tissues from 185 mammalian species that has focused attention on H3K27me3-marked PcG domains. CpGs that consistently gain methylation with age predominantly reside in PRC2-binding sites [63]. The gain of methylation at these sites could be explained by an age-dependent loss of an interaction between PRC2 and Tet dioxygenases [35,45,46]. Loss of Tet-mediated demethylation would result in gain of CpG methylation at PRC2 binding sites. Remarkably, around 90% of the genome-wide age-dependent CpG methylation gain can be accounted for by methylation enriched at PRC2-binding sites, an observation that has enabled the design of a universal biomarker for aging and rejuvenation termed AgeIndex [64].

Linear models predict based on linear relationships between features. A range of models are available that introduce non-linearity between features. AltumAge is a deep neural network based on a 5-layer perceptron where 32 nodes (“neurons”) are connected to learn more complex non-linear CpG-CpG interactions using 142 publicly available data sets [65]. It is an accurate age predictor, outperforming linear models. Using a game-theoretic Shapley-values-based (SHAP) approach for model interpretation, it was possible to measure the importance of CpG sites to the predicted eAge output of AltumAge. CpG sites were assigned SHAP importance values. Intersecting an 18-state Chromatin Hidden Markov Model (ChromHMM) with CpG SHAP importance values showed that SHAP importance values were significantly impacted by ChromHMM state. Interestingly, the chromatin state with the highest SHAP normalized median importance were KRAB-ZNF HLDs followed by the heterochromatin state [65]. As explained, homotypic (self–self) interactions between the KRAB-ZNF HLDs on chromosome 19 emerge in contact frequency (Hi-C) maps as the B4 sub-compartment (Figure 2; [29]); HLDs have a measurable tendency for homotypic interactions and segregate away from the euchromatic “blocks”. This tendency might explain what drives important CpG-CpG interactions that contribute to eAges predicted by non-linear models.

The segregation of HLDs is similar to the behaviour of di-block co-polymers (di-BCPs; see [66] for a review). Di-BCPs consist of a series of alternating blocks (e.g., A-type and B-type), each composed of multiple monomers (A monomers and B monomers). Where the monomers are incompatible the blocks segregate on the basis of like-with-like, with A-type blocks associating with A-type blocks and B-type associating with B-type. Accordingly, the BCP forms spatially segregated domains that are enriched in A or B. Treating chromosomes as BCPs has provided a theoretical framework for understanding the biophysical mechanism(s) that underpin contact enrichments observed in Hi-C maps [67,68,69], which leads us to our next section.

### 1.3. Polymer Physics: The Flory–Huggins Parameter χ

In *Drosophila*, a block co-polymer model consisting of A- and B-type repeating units that assemble euchromatic A- and heterochromatic B-type chromatin, respectively, was used to explain the effects of the HP1a “knock-down” on Hi-C maps if the HP1a bound to H3K9me3 in the B-type heterochromatic compartments causes preferential interactions between heterochromatic B-type chromatin [70]. A similar conclusion was drawn from spectral decomposition applied to Hi-C data from human HCT116 cells that showed the heterochromatic interaction profile group B4 (marked by H3K9me3 and HP1α/β) had the strongest compartmentalization [19].

Applying BCP theory to microphase separation of H3K9me3-marked HLD/Cs from euchromatic “blocks” implies there is a value for the thermodynamic Flory–Huggins parameter, χ [71,72]. χ specifies the *degree of incompatibility* between HLD/Cs and the euchromatic “blocks” and is ultimately the driving force for their segregation from each other [66]. Addressing this issue directly, a crude estimate of χ has been determined from liquid Hi-C experiments [73]. Liquid Hi-C showed, first, that compartmentalization observed in Hi-C experiments is stable when chromatin fragments were 10–25 kb but not when <6 kb, whereupon A and B compartment phase separation is unstable. Next, dissociation kinetics of digested chromatin showed that the interaction of heterochromatic H3K9me3-marked B-type fragments associated with HP1α/β were more stable than those B-type fragments associated with H3K27me3-marked PcG domains indicating that H3K9me3-marked HLD/Cs drive strong B-type compartmentalization in mammalian cells [73], which is consistent with the *Drosophila* studies [70]. Finally, assuming that controlled enzymatic digestion “frees” HLD/Cs and euchromatic “blocks” from the constraints of linear chromosomes resulting in a solvent-free polymer melt, polymer theory was used to estimate χ as 0.20 ± 0.07/kb, measured in units of thermal energy, k_B_T (1 k_B_T = 0.6 kcal/mol at physiological temperatures) [73]. The positive sign of χ indicates that HLD/Cs and the euchromatic “blocks” have a tendency to spontaneously self-associate and segregate away from each other.

## 2. A Biophysical Approach for Estimation of χ Consistent with the Nuclear Environment: Changes in χ During Ageing and OSKM-Driven Age Reprogramming

Polymer theory used to estimate of χ for HLD/Cs vs. euchromatin [73] required some (gross) assumptions: the polymer chains are flexible governed by Gaussian statistics, and they exist in a solvent-free molten state. These assumptions are far removed from the situation in the nucleus where semi-flexible nucleosome fibres exist as a concentrated solution dissolved in the nucleoplasm, which makes simple application of polymer theory for the determination of χ in vivo questionable. To explore the relationship between χ and eAge further, an approach that will estimate χ for HLD/Cs and PcG domains that approximates more closely to their nuclear environment is needed. We have described such an approach [17]. Here, the *unit of incompatibility* is a dynamic multicomponent structure, the oligo-nucleosomal “clutch” of between two and ten nucleosomes; H3K9me3- and H3K27me3-marked nucleosomal arrays form “clutches” when incubated with their corresponding “reader” proteins (Figure 1c,f; [11,25,26]). A euchromatic “clutch” consists of two to ten nucleosomes that are disorganized with weak zig-zag geometry. A heterochromatic “clutch” that makes up an HLD is a repeating unit of two to ten H3K9me3-marked nucleosomes “bridged” by HP1 dimers that compact and stabilise the zig-zag geometry of nucleosomes. Heterochromatic “clutches” are present in the nucleus. ChromHL, a chromatin hierarchical lattice framework, revealed heterochromatin “nanodomains” in murine ES cells, consisting of “clutches” of 3-10 H3K9me2/3-marked nucleosomes bound by HP1 [74]. Our approach utilised an empirical relationship used to determine χ for chemical di-BCPs (see Equation (4) in reference [66]). Accordingly, the magnitude of χ for a heterochromatic “clutch” vs. euchromatic “clutch” can be written using the “clutch” equation as follows (see [17] for details):

**χ_HC_ = [(H_CD-H3K9me_)_HC_ + (H_CSD-CSD_)_HC_ − H_COMP_ − H_TL_]T^−1^ + S_COMP_** (1)

**χ_HC_** denotes χ for a heterochromatic “clutch” of H3K9me3-marked nucleosomes “bridged” by HP1 dimers [17]. The nucleosomes are H1-containing, i.e., chromatosomal [75]. The temperature (**T**) dependent enthalpic component in the square brackets describes the sum of free energy contributions from (i) binding of the HP1 CD to H3K9me3 (**H_CD-H3K9me_**) and homodimerization of the HP1 CSD (**H_CSD-CSD_**) and (ii) the competing elastic energy **H_COMP_** due to linker DNA (i.e., resistance to bending, twisting, stretching deformation of linker DNA and steric exclusion between the nucleosomes and linker DNA) and **H_TL_** elastic energy of the terminal linker DNAs that connects the heterochromatic clutch to a euchromatic clutch. The entropic component **S_COMP_** is the free entropy given up to the nucleoplasm after “bridging” of H3K9me3-marked nucleosomes by HP1 dimers, which stabilises the zig-zag geometry resulting in compaction.

Equation (1) can be used to estimate χ for any “reader” protein that “bridges” modified nucleosomes in a “clutch”, such as the MPP8 reader whose CDs homodimerize and are capable of “bridging” two H3K9me3-marked nucleosomes, like HP1 dimers [76]. It can also be used to estimate χ_PC_ for a “clutch” of H3K27me3-marked nucleosomes “bridged” by CBX2, although it must accommodate the difference in how nucleosomes in the “clutch” are “bridged”. For determination of χ_PC_ using Equation (1), the binding energies of the CaPS (“bridging”) interaction with nucleosomes (Figure 1d,f; [25,26]), and that of the CBX2 CD for H3K27me3 would need to be estimated. The former is not known, but a clue to the magnitude of the latter comes from measurement of the affinity of the CBX2 CD for H3K27me3. The affinity of the CBX2 CD for H3K27me3 is significantly (~40 fold) weaker than the affinity of HP1 CD for H3K9me3 [77]. The weaker affinity indicates the value of χ_PC_ will be lower compared to a HP1 “clutch” χ_HC_. Consistent with χ_PC_ < χ_HC_ compartmentalization of H3K27me3-marked PcG domains is less stable compared to H3K9me3-marked HLD/Cs [19,73,78].

H3K9me3-marked HLD/Cs and H3K27me3-marked PcG domains are dynamic macromolecular assemblies subject to epigenetic drift, a hallmark of ageing [51]. The H3K9me3 modification shows a general decline during aging [51,79]. H3K9me3-marked HLD/Cs are also eroded by loss of fidelity [80] owing to their dis/re-assembly during mitosis and the dynamic nature of “reader” proteins. Fluorescence recovery after photo-bleaching shows that the entire pool of HP1 turns over in around 10s in regions outside constitutive heterochromatin [81] where HLD/Cs reside; constant dynamic exchange of bound with free HP1 dimers in the nucleoplasm helps retain compaction. During mitosis, the bulk of HP1 proteins are removed from chromatin and re-associate in the following interphase [82]. Disruption of H3K9me3-marked HLD/Cs also takes place during DNA replication [83]. The H3K27me3 modification shows a more complex pattern where there is a “re-localization” of H3K27me3 as cells age. In old cells, H3K27me3 is lost from peak regions found in young cells and is gained at lamin-associated domains [84]. The CBX2 H3K27me3 “reader” is, like HP1, highly dynamic in interphase nuclei with a fluorescence recovery time around 10–20s [85]. Unlike HP1, CBX2 becomes tightly bound to mitotic chromosomes at metaphase [85,86]. H3K27me3-marked PcG domains are disrupted during DNA replication [83].

Changes in the levels and distribution of histone modifications and loss of fidelity of HLD/Cs and PcG domains, especially in dividing cells, will affect “bridging” of H3K9me3- and H3K27me3-marked nucleosomes in “clutches” by their respective “readers”. Reduction in histone modifications and binding of “readers” would lead to a decrease in the magnitude of χ_HC_ and χ_PC_. For χ_HC_, the magnitude of **(H_CD-H3K9me_)_HC_ + (H_CSD-CSD_)_HC_** will fall along with **S_COMP_** because compaction is disrupted (see Equation (1)). As a consequence, the number of configurational states assumed by the H3K9me3-marked nucleosomal chain will increase as will combinatorial entropy with surrounding euchromatin because the different chromatin “blocks” become more compatible; the same would happen as χ_PC_ falls. The chromatin “blocks” will begin to mix. This is observed in Hi-C maps as cells age. A/B compartmentalization is reduced, along with increased compartment switching from heterochromatic B-type compartments to euchromatic A-type compartments [87]. Epigenetic rejuvenation by OSKM-driven age reprogramming can restore H3K9me3 levels in old cells to that found in young cells [53,54,55,56,57]. A prediction would be that χ_HC_ would increase as epigenetic rejuvenation restores H3K9me3 levels and more HP1 dimers bind, whereupon HLDs and euchromatic “blocks” would de-mix resulting in an increase in (sharper, more discrete) A/B compartmentalization.

## 3. OSKM-Driven Age Reprogramming vs. Physiological Age Reprograming: Relationship Between χ, eAge and Shannon Entropy

OSKM-driven age reprogramming is an experimental paradigm having great potential for regenerative therapies that will alleviate human suffering (Figure 3a). Biotech has invested billions of dollars in this technology [49,50]. Epigenetic rejuvenation is necessary for OSKM-driven age reprogramming [55,59]. Epigenetic clocks have been used to measure epigenetic rejuvenation quantitatively and shown that eAge declines after introduction of OSK(M) gene activity into old cells [54,58,88,89], while maintaining differentiated cellular identity, even though cells can undergo transient de-differentiation [54]. These observations led to the suggestion that epigenetic rejuvenation by OSKM-driven age reprogramming restores the youthful cell type-specific epigenotype equivalent to that found at the time the differentiated lineage first arose during development; the epigenotype is rejuvenated while retaining differentiated cellular identity [49,59,90]. Although this may be true for OSKM-driven age reprogramming, the situation is different for *physiological* age reprogramming during embryogenesis.

A similar process to OSKM-driven age reprogramming operates during mammalian development (“physiological” age reprogramming [49]; Figure 3b). During pre-implantation/early post-implantation development the hallmarks of ageing, telomere attrition, DNA repair/genomic instability, mitochondrial dysfunction and epigenetic drift are reset to ensure the embryo is optimally primed to direct development (Figure 3b). Epigenetic rejuvenation resets the repressive epigenetic modifications, H3K27me3, CpG methylation and H3K9me3, where maximal levels are reached at embryonic days e6.5 (H3K27me3 and CpG methylation; [98,99]) and e8.25 (H3K9me3; [96]), in or around gastrulation (Figure 3b). These observations indicate the strategy employed by the mammalian embryo for epigenetic regulation of development is to first establish a global “closed” repressive epigenetic environment with repression being more extensive in mesoderm and endoderm compared to ectoderm [96,101], which is “primed” for lineage commitment earlier than the other two germ layers [101]. As cells enter the phylotypic progression [102], at around e8.5, cells are first specified then determined, whereupon they are committed to follow any one of several different cell lineages. Here, physiological age reprogramming differs from OSKM-driven age reprogramming because the former concerns lineage commitment, and the latter rejuvenation of differentiated cells.

During lineage commitment the global repressive epigenetic landscape undergoes a profound reorganization (Figure 3b), where there is loss of repressive modifications at lineage-specific genes and packaging of lineage-inappropriate genes into repressive H3K9me3-marked HLD/Cs [20,96,103] and H3K27me3-marked PcG domains [98,104,105]. DNA demethylation of enhancers by Tet dioxygenases also activates lineage-specific expression during the phylotypic progression [106]. The H3K9me3-marked HLD/Cs and H3K27me3-marked PcG domains assembled at lineage-inappropriate genes safeguard cellular identity. They are “pristine”, having the highest lineage-specific values for χ_HC_ and χ_PC_ because they have not been subject to age-related epigenetic drift (Figure 3b). The entire period from resetting of repressive histone (H3K27me3 and H3K9me3) and DNA (meCpG) modifications up to and including the phylotypic progression (encompassing days e6.5 to e10.5) overlaps with the eAge “ground state”—an eAge minimum that lies between e4.5 and e10.5, with the minimum reached around days e6.5/e7.5 [100]. Putting it short, during the phylotypic progression χ is at its maximum whilst eAge is at a minimum (Figure 3b), indicating a simple negative monotonic relationship between χ and eAge. Assuming a linear relationship:

(2) $$\chi \propto {eAge}^{-1}$$

“Ticking” of the epigenetic clock resumes after day e10.5 [100] and continues as eAge increases during development into adulthood [48,61]. Equation (2) holds true throughout aging, albeit the rate of “ticking” is dependent upon tissue type [61]. As eAge rises, epigenetic drift causes age-dependent changes in the levels/distribution of histone modifications and loss of fidelity of the “pristine” H3K9me3-marked HLD/Cs and H3K27me3-marked PcG domains (where the magnitude of χ_HC_ and χ_PC_ is large), leading inexorably to a “smoothing” out of the epigenetic landscape (where the magnitude of χ_HC_ and χ_PC_ decreases).

Another consequence of epigenetic drift concerns the information content—the specific epigenetic states—stored in HLD/Cs and PcG domains. The function of H3K9me3-marked HLD/Cs and H3K27me3-marked PcG domains is to safeguard cellular identity by silencing lineage inappropriate gene expression in committed cells as they exit the phylotypic progression (Figure 3b). The specific epigenetic states sequestered within the HLD/Cs and PcG domains ensure that the pattern of gene expression of a particular lineage is appropriate for that lineage and, importantly, the epigenetic states are faithfully (epigenetically) inherited from one cellular generation to the next as committed cells divide and expand to populate their respective tissues, including tissue stem cell niches. Put another way, the “signal” sent from one cellular generation (the “sender”) to the next (the “receiver”) should have low uncertainty (low Shannon entropy [107]) to safeguard lineage-specific gene expression. This is initially achieved through the assembly of “pristine” HLD/Cs and PcG domains possessing the highest lineage-specific values for χ_HC_ and χ_PC_ (Figure 3b). The epigenetic specificity stored in HLD/Cs and PcG domains is gradually eroded and lost owing to epigenetic drift. Thermodynamically, as χ_HC_ and χ_PC_ fall in ageing cells HLD/Cs and PcG domains become more compatible with euchromatin, the specificity associated with HLD/Cs and PcG domains is lost, and combinatorial entropy increases. These arguments, we suggest, indicate a relationship between thermodynamic entropy and Shannon entropy that can be stated in terms of the thermodynamic parameter χ and Shannon entropy. In communication theory, the information content in a “signal” sent from “sender” to “receiver” is given in terms of Shannon entropy [107]. Put simply, a signal that has low Shannon entropy has low uncertainty; high Shannon entropy signal has high uncertainty. This allows for a simple negative monotonic relationship between χ and Shannon entropy. Assuming a linear relationship:

(3) $$\chi \propto {Shannon\;Entropy}^{-1}$$

## 4. Perspectives

Our synthesis of epigenetics, machine learning and polymer physics has revealed two novel relationships involving the thermodynamic Flory–Huggins parameter, χ: χ _∝_ eAge^−1^ and χ _∝_ Shannon entropy^−1^. To explore these relationships further, especially during the predicted decay of χ with age and its restoration by epigenetic rejuvenation, an estimate of the magnitude of χ in nuclei is needed. The “clutch” equation (Equation (1)) can be used to estimate χ for any “reader” protein that “bridges” modified nucleosomes in a “clutch”. Equation (1) was first applied theoretically [17] to estimate χ_HC_ for an idealised “clutch” of six H3K9me3-marked nucleosomes “bridged” by HP1 dimers; six nucleosomes lie in the middle of two to ten nucleosomes, which was assumed to be the range of H3K9me3-marked heterochromatic “clutches” in the nucleus. Soon after, it was shown that “clutches” (nanodomains) of 3-10 H3K9me3-marked nucleosomes “bridged” by HP1 are present in interphase nuclei [74]. Because the enthalpic and entropic terms of Equation (1) are dependent on “clutch” size, the magnitude of χ_HC_ will vary according to “clutch” size. Accordingly, heterochromatic “clutches” in the nucleus will have a range of values for χ_HC_. Notably, in vitro studies using purified proteins have estimated the binding energy of the HP1 CD “aromatic cage” to a H3K9me3 peptide [108], indicating that information on the enthalpic component **(H_CD-H3K9me_)_HC_** in Equation (1) is forthcoming and a value for χ_HC_ within reach. Calculating χ_HC_ for different “clutch” sizes in vitro and then intersecting those data with the number and size of clutches in the genome based on a ChromHL framework [74], a range or average value for χ_HC_ for nuclei can be estimated. It would then be possible to test whether the value of χ_HC_ in cells leaving the phylotypic progression is higher than for old cells of the same lineage and, if so, whether χ_HC_ could be restored after epigenetic rejuvenation by OSKM-driven age reprogramming. As a note of caution, the estimation of χ in vivo could be confounded by the observation that many proteins are now known to be associated with H3K9me3-marked heterochromatin [109] that could act in addition to [110] or, indeed, substitute for HP1 proteins as alternative H3K9me3 “readers” (e.g., MPP8; [76]). Alternative H3K9me3 readers, or intrinsic nucleosome condensability [18], might mediate de novo re-compartmentalization of H3K9me3-marked domains at the mitosis-to-G1 phase transition where HP1 function does not appear to be required [111].

Epigenetic rejuvenation reverses epigenetic drift. It restores H3K9me3 levels [53,54,55,56,57] and eAge declines [54,58,88,89]. Recovery of H3K9me3 levels would lead to greater binding of HP1 dimers and increase the value of the enthalpic term **(H_CD-H3K9me_)_HC_ + (H_CSD-CSD_)_HC_** in Equation (1). The magnitude of χ_HC_ would increase. As χ_HC_ is restored, the information content associated with HLD/Cs is also recovered (applying Equation (3)). The same would hold true for χ_PC_ and PcG domains. Epigenetic rejuvenation of old cells by OSKM-driven age reprogramming can then be seen as returning HLD/Cs and PcG domains to their youthful chromatin states with high χ and information content. Cellular identity is safeguarded and, further, buffered against environmental and cellular signalling events that could affect the integrity of the stored information: information is made more resistant to noise. This view is distinct to that set out in the information theory of ageing [112], where emphasis is placed on a hypothetical “backup copy” of youthful information in each (aged) cell that is accessed during epigenetic rejuvenation. Instead, epigenetic rejuvenation reconstitutes that which was eroded by epigenetic drift, such as erosion of HLD/Cs and PcG domains. Reconstitution likely requires chromatin remodelling proteins such as topoisomerase IIα that is necessary for OSKM-driven partial reprogramming [113] and regulates formation of PcG domains [21].

There is evidence that the proximal cause of the “ticking” of “epigenetic clocks” in tissue stem cells and their progeny in part results from epigenetic drift of chromatin-template dependent pathways such as H3K9me3-marked HLD/Cs and H3K27me3-marked PcG domains. Density-based regional epigenetic clocks using age-dependent changes in CpG methylation over a region rather than individual CpGs to predict eAge identified heterochromatin as being enriched for CpGs that lose methylation with age [114]. An epigenetic clock that accounts for non-linear relationships between CpGs (CpG-CpG interactions) indicates that CpGs important for eAge prediction are associated with large HLDs [65]. Evidence that “ticking” of an eAge clock can be driven by erosion of H3K27me3-marked PcG domains is on firmer footing. The gain of DNA methylation at PRC2-rich sites has been used to predict eAge [64], which can be explained by an age-dependent erosion of PcG domains that disrupts PRC2-dependent recruitment of Tet dioxygenases [33,45,46]. Studies have shown that Shannon entropy associated with the methylome increases with age [115]. Because of cross-talk, the increase may shadow the increase in Shannon entropy associated with chromatin-dependent pathways such HLD/Cs and PcG domains owing to the decay of χ (applying Equation (3)) resulting from epigenetic drift.

## 5. Conclusions

Building on a framework for the estimation on of χ in the native nuclear environment [17], we have derived two relationships: $\chi \propto {eAge}^{-1}$ and $\chi \propto {Shannon\;Entropy}^{-1}$. Tests of the relationships can be suggested:

1. $\chi \propto {eAge}^{-1}$. The sign and magnitude of χ_HC_ and χ_PC_ ultimately drives micro-phase separation and segregation of HLD/Cs and PcG domains away from euchromatin. The clutch equation (Equation (1)) predicts that as H3K9me3 and H3K27me3 levels decline (while eAge increases), χ_HC_ and χ_PC_ for HLD/Cs and PcG domains, respectively, decrease leading to a reduction in A/B compartmentalization along with increased A/B compartment switching. This has been observed as cells age. When H3K9me3 and H3K27me3 levels are restored during OSKM-driven epigenetic rejuvenation, χ_HC_ and χ_PC_ increase resulting in de-mixing of HLD/Cs and PcG domains away from euchromatin. Experimentally this would be observed as enhanced (sharper, more discrete) A/B compartmentalization. Use of specific inhibitors of HMTases that generate H3K9me3 and H3K27me3 would confirm that compartmentalization is driven by restoration of the epigenetic histone modifications.
2. $\chi \propto {Shannon\;Entropy}^{-1}$. This can be tested indirectly using, as a measure, Shannon entropy associated with the methylome. The clutch equation (Equation (1)) predicts that as H3K9me3 and H3K27me3 levels decline (while eAge increases) χ_HC_ and χ_PC_ for HLD/Cs and PcG domains, respectively, will decrease. This leads to increased disorder, i.e., increased combinatorial entropy. Given the known cross-talk between chromatin-template-dependent pathways (HLD/Cs and PcG domains) and the DNA de/methylation machinery the increase in disorder of chromatin-template dependent pathways can be measured by an increase in Shannon entropy associated with the methylome. Restoration of H3K9me3 and H3K27me3 levels by OSKM-driven epigenetic rejuvenation will increase χ_HC_ and χ_PC_ for HLD/Cs and PcG domains that can be measured by a reduction in Shannon entropy associated with the methylome. Use of specific inhibitors of HMTases that generate H3K9me3 and H3K27me3 would confirm that reduced Shannon entropy of the methylome is driven by restoration of the epigenetic histone modifications.
